# Carbolic Acid in Piles and Other Diseases

**Published:** 1883-06-20

**Authors:** J. G. Westmoreland

**Affiliations:** Atlanta, Ga.


### Southern Medical Record:

EDITORS:

T. S. POWELL, M.D. W. T. GOLDSMITH, M.D. R. C. WORD, M.D.

*R. C. WORD, M.D., Managing Editor.*

☞All Communications and Letters on Business connected with the RECORD must be addressed to the Managing Editor.

VOL. XIII. ATLANTA, GA., JUNE 20, 1883. No. 6.

### ORIGINAL AND SELECTED ARTICLES.

### CARBOLIC ACID IN PILES AND OTHER DISEASES.

BY J. G. WESTMORELAND, M. D., ATLANTA, GA.

In the SOUTHERN MEDICAL RECORD for May will be found a short article on the treatment of piles by carbolic acid. In it little more was stated than the mere fact of injection of the hemorrhoidal tumor with the undiluted acid.

Now, in compliance with the request of a reader of the article, I propose to give a more extended review of the subject, and the application of the remedy in the treatment of other local diseases.

From time to time articles have appeared in which I have tried to impress members of the profession with the peculiar and useful action of carbolic acid—its cathartic or local alterative, and hæmostatic effects. In medical society discussions, while giving my experience of its safety and comparative freedom from pain when applied to diseased mucous membranes, I have found myself opposed by almost every member, and as most of them refused to use it as recommended, I was generally left without proof of my position.

Shortly after the remedy had been brought to notice, I, from some report of its action probably, used it as a general styptic for menorrhagia, in the dose of two drops repeated every two hours.

Proving successful for the relief of this hemorrhagic condition I decided to test it in purpura hemorrhagica. Accordingly, in a case presented in the Medical College Clinic, which had been treated without benefit by the usual means—muriated tincture of iron, etc., I prescribed carbolic acid in the dose of two or three drops every three or four hours, well diluted with water, with complete success in a day or two.

While the styptic effect may not be universally recognized, no one, as far as I know, objects to its use in this way. It is the local action, as described, which calls forth the opposition. When it is asserted that the undiluted acid may be applied to chancre, chancroid, diphtheria, chronic intra-uterine inflammation, etc., with very little pain, the war begins. Physicians assert—of course without ever having tested it—that destructive action will be had upon the mucous membrane and therefore cicatricial tissue necessarily follows. Learned gynecologists, to whose use the remedy seems to me peculiarly adapted, forbid its introduction into the uterus, alleging that stenosis will be the result. Whether this conclusion is reached by experience or reasoning, it is certainly not more reliable than the opinion formed by hundreds of applications of the pure acid made in endo-metritis, diphtheria, cancerous ulcers, etc., without stenosis or other unfavorable result. When properly applied so as to come in contact with the diseased part alone, or the internal mucous membrane, very slight pain is produced, not more than that from astringent solutions of moderate strength. I have applied it with a camel's hair pencil to the pseudo-membrane of diphtheria in the mouth of an infant, and by injection to the urethra in gonorrhœa, taking care that the acid does not come in contact with the lips, glans penis or prepuce. When applied to the uterus or vagina, its contact with the pudendum must be avoided.

In the treatment of cancer I have used the acid only as an application to the ulcerated surface, but from the promptness with which progress is arrested, and superficial healthy tissue formed, I have hope of success, to some extent at least, in the bold attempt at cure by injection into the indurated tumor.

In the commencement of what seemed to be malignant (cauliflower) disease of the os uteri, complete and permanent relief has followed the twice a week application of pure acid.

Piles may be permanently cured in half a week or two weeks by injecting the tumor with undiluted carbolic acid. The needle of a hypodermic syringe charged with the acid, must be plunged into the center of the tumor and the piston slightly moved forward so as to discharge one to three drops. Let the needle remain for a minute and then withdraw. Each tumor must be thus injected, and if large two punctures should be made. The tumor becomes pale, shrivels and generally becomes dark and putrid in a day or two. No great pain attends the operation. Only a stinging sensation is experienced, which lasts a few moments.

When several tumors exist a second operation sometimes becomes necessary, owing to the failure to inject all the prominent points. On the second day the injection may be repeated, if all the tumors do not become pale, lessened in size or dark.

The benefit derived from carbolic acid is, doubtless, derived from the peculiar action upon the capillaries and the blood itself. When applied to a surface, it becomes white and bloodless, and when thrown into a mass of blood it is made more or less coagulable, according to the proportion of the mixture. This driving of the blood, as it were, from the surface, accounts for the control of inflammation by its local application, and the coagulating quality renders it useful as a general styptic. Indeed, inflammation, I believe, may be prevented, to a large extent, in a part injured, by a proper application in diluted form. And, while its good effects in this way are attributed to the destructive action upon bacteria, we think its good effects can be explained without recognizing the existence of these animalculæ as factors in the production of inflammation and putrescence.

